# Sex-Specific Patterns of Body Mass Index Relationship with White Matter Connectivity

**DOI:** 10.3233/JAD-215329

**Published:** 2022-04-19

**Authors:** Farzaneh Rahmani, Qing Wang, Nicole S. McKay, Sarah Keefe, Nancy Hantler, Russ Hornbeck, Yong Wang, Jason Hassenstab, Suzanne Schindler, Chengjie Xiong, John C. Morris, Tammie L.S. Benzinger, Cyrus A. Raji

**Affiliations:** a Mallinckrodt Institute of Radiology, Division of Neuroradiology, Washington University in St. Louis, St. Louis, MO, USA; b Department of Neurology, Washington University in St. Louis, St. Louis, MO, USA; c Charles F. and Joanne Knight Alzheimer Disease Research Center (Knight ADRC), Washington University, St. Louis, MO, USA

**Keywords:** Aging, Alzheimer’s disease, body mass index, connectome, diffusion magnetic resonance imaging, white matter

## Abstract

**Background:**

Obesity is an increasingly recognized modifiable risk factor for Alzheimer’s disease (AD). Increased body mass index (BMI) is related to distinct changes in white matter (WM) fiber density and connectivity.

**Objective:**

We investigated whether sex differentially affects the relationship between BMI and WM structural connectivity.

**Methods:**

A cross-sectional sample of 231 cognitively normal participants were enrolled from the Knight Alzheimer Disease Research Center. Connectome analyses were done with diffusion data reconstructed using q-space diffeomorphic reconstruction to obtain the spin distribution function and tracts were selected using a deterministic fiber tracking algorithm.

**Results:**

We identified an inverse relationship between higher BMI and lower connectivity in the associational fibers of the temporal lobe in overweight and obese men. Normal to overweight women showed a significant positive association between BMI and connectivity in a wide array of WM fibers, an association that reversed in obese and morbidly obese women. Interaction analyses revealed that with increasing BMI, women showed higher WM connectivity in the bilateral frontoparietal and parahippocampal parts of the cingulum, while men showed lower connectivity in right sided corticostriatal and corticopontine tracts. Subgroup analyses demonstrated comparable results in participants with and without positron emission tomography or cerebrospinal fluid evidence of brain amyloidosis, indicating that the relationship between BMI and structural connectivity in men and women is independent of AD biomarker status.

**Conclusion:**

BMI influences structural connectivity of WM differently in men and women across BMI categories and this relationship does not vary as a function of preclinical AD.

## INTRODUCTION

Obesity is linked to microstructural white matter (WM) alterations [1, 2]. These effects become increasingly prominent at midlife, when higher total body fat and waist circumference translate to increased fractional anisotropy (FA) and lower mean diffusivity (MD) of WM fibers [1–4]. Overweight adults demonstrate decreased WM connectivity, lower fiber density, and reduced FA in fibers connecting different parts of the taste-reward network [5–7]. Several studies point to alterations in brain connectivity in the homeostatic and reward networks of the brain that control the reflexive and behavioral aspects of eating and hence contribute to the pathogenesis of obesity [8]. These alterations, whether they cause or are being caused by obesity, are prominent to the extent that patterns of structural WM connectivity can differentiate the brains of overweight young adults from those with a normal weight with high accuracy [5].

Obesity is associated with neuroinflammation [9, 10]. High body fat and high fat diet lead to increased levels of proinflammatory cytokines and immune cells which in turn result in blood-brain barrier dysfunction that gives rise to central inflammation and endothelial dysfunction [9]. Neuroinflammation is itself associated with reduced WM integrity and axonal density [11], an effect that is mediated by and accentuated through endothelial dysfunction, blood-brain barrier dysfunction, and the resulting demyelination [12, 13]. Higher body mass index (BMI) and systemic inflammation can also affect WM myelin content, and WM integrity as a result, through mechanisms independent of peripheral or central inflammation [14–16]. Obesity can also accentuate the age-related structural decline in certain WM fibers [17]. Neuroinflammation and blood-brain barrier dysfunction are also increasingly investigated mechanisms in Alzheimer’s disease (AD) [18, 19], which itself is associated with mid-life obesity [20]. Obesity itself modifies the effect of neuroinflammation on hippocampal atrophy and amyloid-β burden which are important biomarkers of AD [20, 21], making it important to investigate the potential modifying effect of AD status on the relationship between obesity and WM connectome.

While sexual dimorphisms in WM connectivity have been examined in prior work [22–24], there are comparatively recent investigations linking lifestyle-related risk factors, such as obesity, diabetes, and hypertension, with sex differences [7, 25, 26]. Biological sex not only alters brain connectivity but also affects energy homeostasis, body fat distribution, and the likelihood of obesity, where women are more likely to be obese but men are more prone to obesity-related chronic diseases [27, 28]. Sex can also differentially affect the life-long trajectory of WM development characterized by more advanced development of WM in preadolescent girls and a steeper slope of age-related changes in WM structure in men [28]. Sex difference in obesity and WM structure can render either the male or female brain more susceptible to the adverse effects of high body fat in later life. While one study points to a higher likelihood of myelin degeneration in WM of obese young women [29], no study has investigated the potential modifying effect of sex on the association of BMI with WM connectome in middle-age to older adults. Thus, the magnitude of obesity driven risk for chronic WM damage in men versus women remains unclear.

We therefore conducted a diffusion MRI correlational connectometry analysis in 231 healthy middle-age and older adults from the Charles F. and Joanne Knight Alzheimer Disease Research Center (Knight ADRC). This study investigates: 1) whether biological sex differentially modifies the relationship between BMI and WM structural connectivity and how this relationship is affected in different BMI categories such as normal weight, overweight, obese and morbidly obese, 2) whether there is an interaction between biological sex and BMI in altering WM structural connectivity, and 3) how do the above relationships change in the context of preclinical AD as a modifier of WM structure in this population given the role of mid-life obesity in AD risk [30].

## METHODS

### Participants

**Fig. 1:**
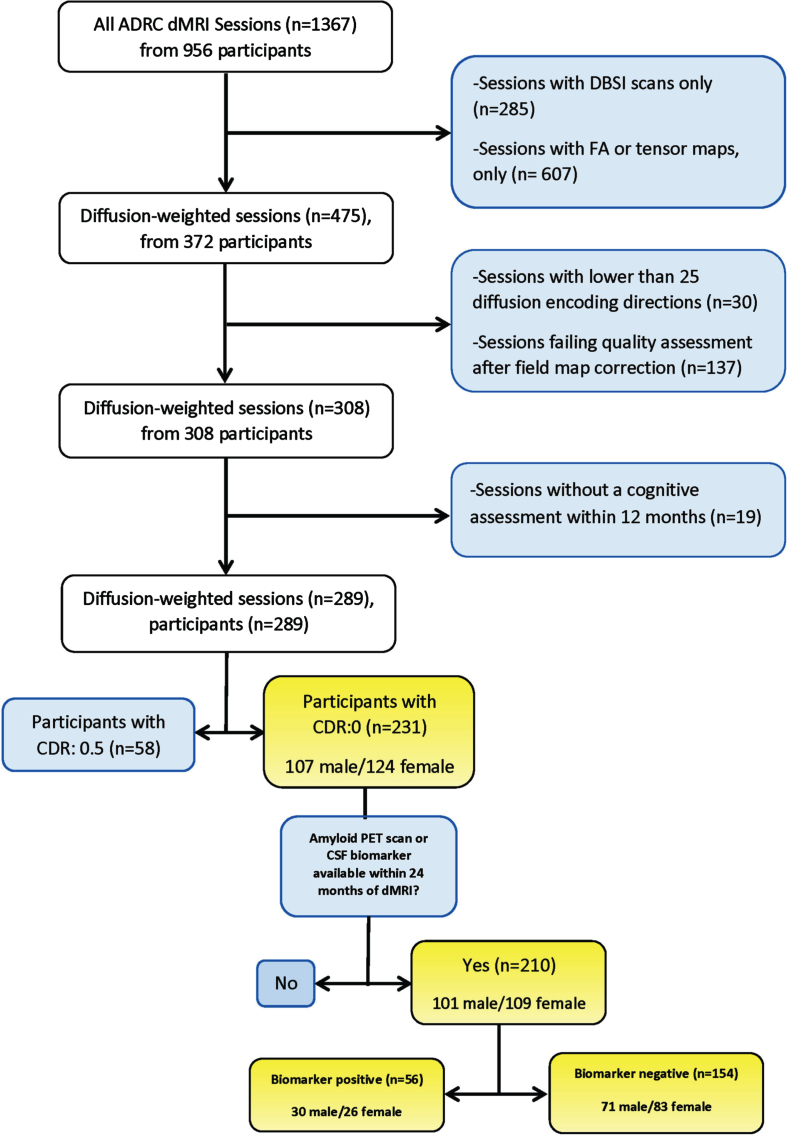
Inclusion and exclusion criteria for the study population and sample size. ADRC, Charles F. and Joanne Knight Alzheimer Disease Research Center; dMRI, diffusion magnetic resonance imaging; CDR, Clinical Dementia Rating; CSF, cerebrospinal fluid; PET, positron emission tomography.

We selected 231 community dwelling individuals (107 men, 124 women; mean age: 69.2±8.3 years, age range: 42–88 years) from a larger cohort of participants enrolled in the ongoing longitudinal studies of memory and aging conducted at the Knight ADRC at Washington University School of Medicine in St. Louis. Figure 1 demonstrates how this cross-sectional sample was determined based on inclusion and exclusion criteria. All Knight ADRC participants with at least one diffusion magnetic resonance imaging (dMRI) session and Clinical Dementia Rating (CDR) score = 0 [31] associated with that session were included. Participants were excluded if they: 1) lacked a cognitive assessment within 12 months of the dMRI scan, or 2) had abnormal cognition based on a CDR > 0 at the assessment closest to the scan. Exclusion criteria for dMRI scans were: 1) having lower than 25 diffusion encoding directions, that could potentially result in the suboptimal generation of the reference local connectome, or 2) low image quality after field map and eddy current correction. The relationship between WM structural connectome and BMI was evaluated separately in men and women, followed by the relationship between WM structural connectome and the interaction between BMI and sex. Age, cognitive scores, and apolipoprotein E epsilon 4 (*APOE* ɛ4) carrier status of the participants were entered separately as covariates in the models, as previous literature suggests a relationship between these variables and WM connectome in adults [32–34]. Participants were also categorized based on their BMI into underweight (BMI < 18.5 kg/m^2^), normal weight (BMI: 18.5–24.9 kg/m^2^), overweight (BMI: 25–29.9 kg/m^2^), obese (BMI: 30–39.9 kg/m^2^), and morbidly obese (BMI≥40 kg/m^2^) categories [35], and the relationship between BMI and WM connectome was further assessed within each of the following BMI subgroups: normal weight, overweight, and obese+morbidly obese. As the number of male and female participants in the morbidly obese subcategory was relatively small, we decided to merge the obese and morbid obese groups in analyses based on BMI categories.

Additionally, amyloid positron emission tomography (PET) or cerebrospinal fluid (CSF) AD biomarkers within 24 months of the dMRI scan were characterized for the majority of our sample (*n* = 210). Among these, participants who had a positive amyloid PET uptake or, if not present, positive CSF AD biomarker, were considered as AD *biomarker positive*. Amyloid PET of CSF AD biomarker positivity were both determined based on established ADRC thresholds [36, 37]. Participants who did not meet these criteria were considered AD *biomarker negative*. As cognitively normal individuals with positive AD biomarkers are characterized as having preclinical AD [38], we investigated BMI relationships separately in the AD biomarker positive and biomarker negative subgroups. The goal of subgroup analyses was to perform a sensitivity testing based on preclinical AD status in this midlife to elderly population.

### Standard protocol approvals, registrations, and patient consents

The institutional ethics review board of Washington University School of Medicine in Saint Louis approved the protocol of this study. The study was done in accord with the ethical standards of the Committee on Human Experimentation of the institution in which the experiments were done or in accord with the Helsinki Declaration of 1975. All participants consented to participate in the study and provided their written informed consent to participate in the study. Participants also provided their consent for the data obtained and used in the study to be published in scientific journals.

### MRI acquisition and preprocessing

Included scans were acquired from January 2010 through December 2019. dMRI data were all acquired on 3T Siemens scanners (TIM Trio, Siemens, Erlangen, Germany and Biograph mMR, Siemens, Malvern, PA) located at Washington University School of Medicine in Saint Louis. dMRI data were acquired through a 2D single-shot echo planar imaging (EPI) diffusion sequence, with TR = 14,500  ms, TE = 112 ms, maximal b-value = 1400 s/mm^2^, in-plane resolution = 2 × 2 mm ^2^, slice thickness = 2 mm, and a field of view = 256 mm. Scans all had 25 diffusion encoding directions. A total of 231 dMRI scans were included in the connectome database. The b-table was checked by an automatic quality control routine to ensure its accuracy [39]. Preprocessing steps for diffusion data included eddy current distortion and field map correction which were performed using the eddy_correct and the FMRIB’s Utility for Geometrically Unwarping EPIs (FUGUE) functions from FMRIB Software Library (FSL) v5.0 software. Motion correction and smoothing were performed as part of dMRI connectometry processing through the DSI Studio software package.

### Amyloid PET acquisition and processing

Amyloid PET imaging was performed using either ^11^C Pittsburgh Compound-B (PiB) or ^18^F-AV-45 (florbetapir). Details of acquisition and postprocessing for PiB and AV45 PET have been described elsewhere [40, 41]. Participants were considered amyloid PET positive if they had partial volume corrected mean cortical standardized uptake values of > 1.42 or > 1.19 for PiB and AV-45 respectively [36].

### CSF collection and analysis

Twenty to thirty milliliters of cerebrospinal fluid was collected via lumbar puncture with a 22-gauge atraumatic spinal needle following overnight fasting. Samples were gently inverted to reduce possible gradient effects and then centrifuged at low speed and stored in 0.5 ml aliquots in polypropylene tubes at –84°C. The concentrations of CSF phosphorylated tau (pTau) and amyloid-β peptide 42 (Aβ_42_) were measured with Elecsys® (Roche Diagnostics) assays [37]. A cut-off value for CSF pTau/Aβ_42_ of > 0.0198 is highly concordant with established cut-off values for amyloid PET status and was used to define biomarker positive status [37].

### Cognitive and physical evaluation

Each dMRI scan was paired with the closest cognitive assessment with the maximum interval being 12 months. The mean interval between the dMRI and cognitive assessment was 84±54 days. The CDR and Mini-Mental State Exam (MMSE) were performed at the same cognitive assessment [42]. The participant’s weight and height on the date of the dMRI acquisition were used to calculate BMI. An interaction term was calculated by multiplying the raw and mean-centered BMI values by participant’s sex.

### Connectome analysis

Connectome analyses were performed using the DSI Studio Software [43]. Diffusion data were reconstructed in the Montreal Neurological Institute (MNI) space using q-space diffeomorphic reconstruction (QSDR) to obtain the spin distribution function (SDF) [44, 45]. To characterize WM connectivity, the SDF was extracted at the atlas fiber orientations as the local connectome fingerprint. A diffusion sampling length ratio of 1.25 was used, and the restricted diffusion was quantified using restricted diffusion imaging with an output resolution of 2 mm isotropic. Quantitative anisotropy (QA) values were calculated at the peak orientations of each SDF function and later were used in the probabilistic tractography analysis [46]. A mask consisting of cerebellar white matter and cerebellar cortex was used as a terminative region to exclude cerebellar white matter from tractography results [47].

### Correlational tractography and statistical analysis

We utilized a semi-automated atlas-based deterministic tracking algorithm to obtain correlational tractography based on Spearman rank-based correlation [48]. Correlational tractography is a tractography model that shows trajectories of pathways correlated with a variable of interest. Connectometry is the method to derive correlational tractography and test its reliability. dMRI connectometry adopts a *tracking the correlation* paradigm, which is fundamentally different from the conventional DTI analysis paradigm of finding the correlation between variables of interest and tract parameters. Connectometry uses a nonparametric permutation test to first identify voxels that have strong association with the study variable and tracks along axonal fiber directions to identify the consecutive voxels that show a continuous strong positive or negative association. We performed correlational tractography between QA and BMI and between QA and BMI interaction with sex. In brief, a non-parametric approach was implemented through the Spearman rank-based correlation to consider the non-linear effect between BMI or BMI^*^Sex and QA. The model consisted of either a simple Spearman’s correlation (with no additional covariates) or a partial Spearman’s correlation considering age, cognitive status (MMSE score), or *APOE* ɛ4 allele carrier status as covariates in the model. Covariates were chosen based on literature demonstrating changes in WM connectome with age and the association of WM connectome with cognitive performance and *APOE* ɛ4 carrier status [32–34]. Given the 12-month interval allowed between the clinical and dMRI assessments, the absolute time difference between the clinical assessment and dMRI acquisition was added as a covariate to all models that included the MMSE score (MMSE as covariate). No false discovery rate (FDR) threshold was adopted to keep or exclude variables from the model. The monotonicity assumption for Spearman’s test was checked through visual inspection of a scatterplot between BMI and QA of a representative sample of tracts (10 from each hemisphere) [49].

Different T-score thresholds including 2, 2.5, and 3 were adopted. Models with T-score threshold of 2.5 yielded the strongest correlation while converging to reliably consistent results. As a result, a T-score threshold of 2.5 and a length threshold of 20 mm were assigned with a FDR threshold of 0.05 used to select tracks through a deterministic fiber tracking algorithm [50]. FDR is different from *p*-value as it has a much higher true positive reporting rate and is less sensitive to sample size. The QA values were normalized prior to the analyses, and tracts were filtered by topology-informed pruning with 4 iterations [51]. To estimate the FDR, a total of 10000 randomized permutations were applied to the group label to obtain the null distribution of the track length and statistics. Once the model converged, an average atlas of tracts meeting the tracking parameters is generated separately for negative and positive correlation results. Tracts were identified and labeled through automated reconstruction based on HCP-842 tractography atlas [52]. Results of each model were reported as FDR and Spearman’s correlation coefficient (rs). An r_s_ between 0.1 and 0.29 is considered to represent a small association, while r_s_ represent a medium and coefficients above 0.5 are considered to demonstrate a large association or relationship.

### Data availability

De-identified participant data including clinical and cognitive assessment, CSF biomarkers and dMRI images are available upon request through the Knight ADRC Leadership Committee (https://knightadrc.wustl.edu/research/resourcerequest.htm). The statistical and image analyses were all done through the DSI Studio (http://dsi-studio.labsolver.org) that is publicly available.

## RESULTS

Figure 1 demonstrates the numbers of participants at each stage of study including the total number of participants assessed for eligibility criteria, participants included in the study and those enrolled in subgroups analyses and analyzed in each group. Table 1 describes the demographic and cognitive status of the study population (Table 1 top panel). There was no statistically significant difference between men and women in BMI or the distribution of BMI categories (underweight, normal weight, overweight, obese, and morbidly obese). Men and women were also comparable in their age, years of education, *APOE* ɛ4 carrier status, self-reported race, systolic and diastolic blood pressure and MMSE score (Table 1).

**Table 1:** Description of clinical and cognitive outcomes of the study population

	All participants enrolled (*n* = 231)		
	Male (*n* = 107)	Female (*n* = 124)	*p*
Age, y (mean±sd)	68.6±8.8	67.9±8.1	0.06
Education, y (mean±sd)	16.8±2.3	15.8±2.6	0.45
*APOE* ɛ4 genotype, C, N, M (n)	43/63/1	46/77/1	0.60
Race, C, AA, A, O (n)	86/18/2/1	93/31/0/0	0.32
MMSE (mean±sd)	28.9±1.4	28.8±2.8	0.73
Systolic blood pressure, mmHg (mean±sd)	130.5±17.8	132±70.3	0.54
Diastolic blood pressure, mmHg (mean±sd)	76.1±10.2	80.5±83.8	0.77
Mean arterial pressure, mmHg (mean±sd)	94.3±11.1	98.7±72.3	0.84
BMI, kg/m2 (mean±sd)	27.3±4.7	28.2±6.6	0.99
BMI categories, U, N, Ow, Ob, Mo (n)	3/27/55/16/6	4/36/42/22/20	0.06
	Participants with available AD biomarkers (*n* = 210)		
	Male (*n* = 101)	Female (*n* = 109)	*p*
Biomarker positive, n (%)	30 (29.7%)	26 (23.8%)	0.338
Age, y (mean±sd)	68.2±8.6	67.9±8.2	0.667
Education, y (mean±sd)	16.8±2.3	15.8±2.5	0.003
*APOE* ɛ4 genotype, C, N, M (n)	42/59	38/71	0.341
Race, C, AA, A, O (n)	80/18/2/1	84/25/0/0	0.268
MMSE (mean±sd)	28.9±1.4	28.8±3	0.927
Systolic blood pressure, mmHg (mean±sd)	130.3±17.9	133.2±74.8	0.125
Diastolic blood pressure, mmHg (mean±sd)	76±10.3	74.1±10.3	0.256
Mean arterial pressure, mmHg (mean±sd)	94.1±11.2	98.8±77.1	0.171
BMI, kg/m2 (mean±sd)	27.4±4.6	27.9±6.5	0.925
BMI categories, U, N, Ow, Ob, Mo (n)	2/23/54/16/6	3/34/38/16/18	0.038
	Participants with available AD biomarkers (*n* = 210)		
	Biomarker positive (*n* = 56)	Biomarker negative (*n* = 154)	*p*
Men/Women (n)	30/26	71/83	0.338
Age, y (mean±sd)	72.8±6.8	66.3±8.2	0.116
Education, y (mean±sd)	16.2±2.7	16.3±2.4	0.538
*APOE* ɛ4 genotype, C, N, M (n)	40/15/1	40/113/1	< 0.001
Race, C, AA, A, O (n)	48/7/1/0	116/36/1/1	0.277
MMSE (mean±sd)	28.2±4.07	29±1.2	0.01
Systolic blood pressure, mmHg (mean±sd)	133.4±19.2	131.2±63.5	0.678
Diastolic blood pressure, mmHg (mean±sd)	74.9±9.6	80.4±66.3	0.451
Mean arterial pressure, mmHg (mean±sd)	94.4±11.3	97.3±65.1	0.464
BMI, kg/m2 (mean±sd)	25.8±4.7	28.4±5.8	0.119
BMI categories, U, N, Ow, Ob, Mo (n)	1/25/23/4/3	4/32/69/28/21	0.06

Top panel: the demographic and cognitive status of the study population; Middle and Bottom panels: the demographic and cognitive status of participants with available AD biomarkers categorized by sex (middle) and biomarker status (bottom). *P*-values below 0.05 are indicated as Bold. *APOE* ɛ4, apolipoprotein E epsilon 4 allele; CDR, Clinical Dementia Rating Scale; CDR-SOB, Clinical Dementia Rating Scale Sum of Boxes Scores; MMSE, Mini-Mental State Examination, where 30 = the “best” and 0 = the “worst” scores; BMI, body mass index; *APOE* ɛ4 genotypes: C, carrier; N, non-carrier; M, missing; Race: C, Caucasian; AA, African-American; A, Asian; BMI categories: U, underweight; N, normal; Ow, overweight; Ob, obese; Mo, morbidly obese; AD, Alzheimer’s disease.

### Male-specific relationship of body mass index with reduced connectivity in long-distance associational fibers of the temporal lobe

**Fig. 2:**
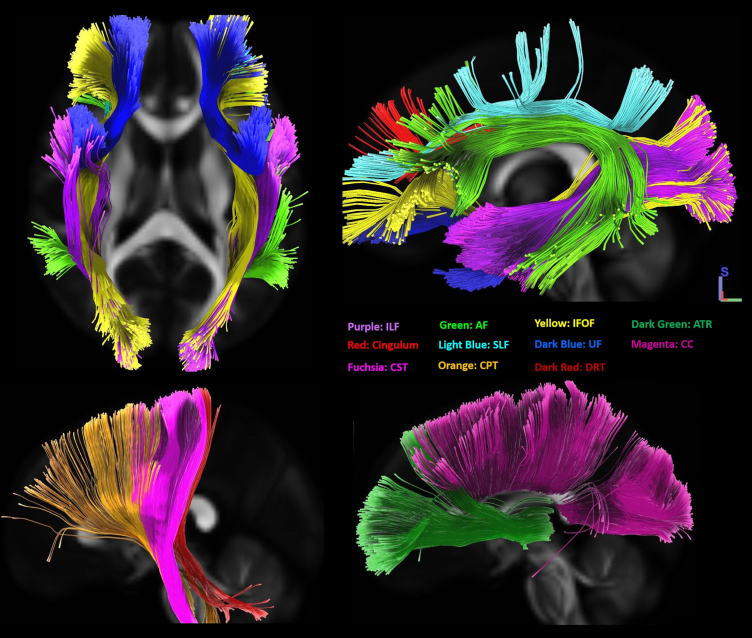
Overview of all significant white matter tracts from the correlational tractography models. ILF, inferior longitudinal fasciculus; AF, arcuate fasciculus; IFOF, inferior frontooccipital fasciculus; ATR, anterior thalamic radiation; SLF, superior longitudinal fasciculus; UF, uncinated fasciculus; CC, corpus callosum; CST, corticospinal tract; CPT, corticopontine tract; DRT, dentatorubrothalamic tract; RST, reticulospinal tract.

Figure 2 is a visual demonstration of all tracts identified through correlational tractography based on the HCP1065 average template [35]. Correlational tractography in men revealed that a higher BMI is related to decreased connectivity in the bilateral corticospinal, corticostriatal, and corticopontine tracts, as well as bilateral inferior longitudinal fasciculi (ILF), bilateral inferior frontooccipital fasciculi (IFOF), and the right frontoparietal part of the cingulum (FDR = 0.04) (Table 2). Connectivity in the tapetum region of the corpus callosum was also negatively related with BMI in men (FDR = 0.02) (Table 2). All statistically significant relationships persisted after adding age as covariate, except for left IFOF (FDR < 0.01). Similarly, MMSE score did not change the negative relationship between BMI in men and connectivity in any of the mentioned white WM tracts (FDR = 0.015). A negative influence between BMI and connectivity in the left parahippocampal area of the cingulum appeared only after removing the effect of age as covariate (FDR = 0.01) (Table 2).

**Table 2:** Summary of models investigating the relationship of white matter connectivity with BMI

Model ^*^	r_S_^§^	Tracts with *negative* relationship of connectivity with BMI in *men*			
No covariate	0.87	•Right and left corticospinal tracts	Left IFOF
Age as covariate	0.71	•Right and left corticopontine tracts	Left parahippocampal cingulum
MMSE as covariate^§§^	0.7	•Right and left corticostriatal tracts	Left IFOF
*APOE* ɛ4 as covariate	0.7	•Right and left ILF	–
		•Right IFOF
		•Right frontoparietal cingulum
		•Tapetum of corpus callosum
Model^*^	r_S_^§^	Tracts with *positive* relationship of connectivity with BMI in *men*			
No covariate	0.58	–
Age as covariate	0.43		Left anterior thalamic radiation	Right dentatorubrothalamic
MMSE as covariate^§§^	0.4		–
*APOE* ɛ4 as covariate	0.55		–
Model^*^	r_s_^§^	Tracts with *negative* relationship of connectivity with BMI in *women*			
No covariate	0.73		–
Age as covariate	0.68		–
MMSE as covariate^§§^	0.65		Left IFOF	Left ILF	Tapetum of corpus callosum
*APOE* ɛ4 as covariate	0.68	–
Model^*^	r_S_^§^	Tracts with *positive* relationship of connectivity with BMI in *women*			
No covariate	0.85	•Right and left frontoparietal cingulum	Left corticospinal	Left dentatorubrothalamic	Left corticopontine
Age as covariate	0.67	•Right and left reticulospinal	Right and left SLF	Right IFOF
MMSE as covariate^§§^	0.7	•Right dentatorubrothalamic	Left dentatorubrothalamic
APOE ɛ4 as covariate	0.61	•Right corticopontine	–
		•Tapetum of corpus callosum
Model^*^	r_S_^§^	Tracts with *positive* relationship of connectivity with BMI^*^Sex			
No covariate	0.47	•Right and left frontoparietal cingulum	–
Age as covariate	0.31	•Right and left parahippocampal cingulum	–
MMSE as covariate^§^	0.33	•Right and left SLF	–
*APOE* ɛ4 as covariate	0.31
Model^*^	r_S_^§^	Tracts with *negative* relationship of connectivity with BMI^*^Sex
No covariate	0.47	•Right and left ILF	–
Age as covariate	0.39	•Tapetum of the corpus callosum	–
MMSE as covariate^§§^	0.20	•Right IFOF	–
*APOE* ɛ4 as covariate	0.38	•Right corticostriatal	–
		•Right corticopontine
		•Right anterior thalamic

^*^All models consisted of a simple or partial Spearman rank-based correlation with local connectivity as dependent variable. Each row demonstrates white matter regions where connectivity was associated with BMI or BMI interaction with Sex (BMI^*^Sex) given the parameters. Male sex was used as reference category in interaction analyses. The false discovery rate threshold was set to 0.05 for all models. ^§^Spearman’s correlation coefficient. ^§§^ The absolute time difference between clinical assessment and diffusion MRI acquisition was used as covariate in any model that included the MMSE score. BMI, body mass index; MMSE, Mini-Mental State Examination; ILF, inferior longitudinal fasciculus; IFOF, inferior frontooccipital fasciculus; SLF, superior longitudinal fasciculus.

Correlational tractography did not reveal any tracts where increased connectivity was associated with higher BMI in men, a result that persisted after removing the effect of participants cognitive score. Removing the effect of age revealed a significant positive relationship between connectivity in the left anterior thalamic radiation and the right dentatorubrothalamic tract and BMI in men (Table 2).

In women, there were no tracts in which lower connectivity was related to higher BMI, a result that persisted after correction for participant age. When adding MMSE score as a covariate in the model, connectivity in the left ILF, left IFOF, and the tapetum of the corpus callosum showed a significant negative association with BMI in these participants (Table 2). Women demonstrated a relationship between higher BMI and increased connectivity in several WM tracts, including the bilateral frontoparietal cingulum, bilateral reticulospinal, dentatorubrothalamic, and corticopontine tracts, as well as the left corticospinal tract and the tapetum area of corpus callosum (FDR < 0.01) (Table 2). Adding participant’s age as a covariate eliminated the effect of connectivity in the left corticopontine and the left dentatorubrothalamic tracts as significant effects (FDR < 0.001 in all models), while revealing a significant positive relationship between connectivity in the bilateral superior longitudinal fasciculi (SLF) and BMI in women (FDR < 0.001 in all models). Adding MMSE score as a covariate to the model removed the significant positive relationship between connectivity in the left corticopontine tract and left corticospinal tracts and BMI in female participants (FDR < 0.001 in all models) (Table 2).

In separate analyses, we investigated tracts where an interaction term between BMI and participant’s sex was related to connectivity (Table 2). Male sex was used as a reference category. A positive association was identified between connectivity of the bilateral frontoparietal and parahippocampal parts of the cingulum and bilateral SLF (FDR < 0.01). These results indicate that as BMI increases, connectivity in these WM fibers tends to be higher in women compared to men. By contrast, connectivity within the right IFOF, bilateral ILF, the tapetum of the corpus callosum, right corticostriatal, right corticopontine, and the right anterior thalamic radiation was negatively associated with the interaction term between BMI and sex (FDR < 0.01). These results indicate that men with higher BMIs have reduced connectivity in these WM tracts compared to women. Adding *APOE* ɛ4 carrier status as a covariate to the model did not change the results of the correlational tractography in neither men nor women (Table 2).

### Higher BMI negatively affects the female white matter connectome only in obese and morbidly obese individuals

We also investigated the association between BMI and white matter connectome in men and women from different BMI subcategories separately. Results of the correlational tractography in normal weight (BMI: 18.5–24.9 kg/m^2^), overweight (BMI: 25–29.9 kg/m^2^), obese and morbidly obese (BMI≥30 kg/m^2^) categories are presented in Table 3. Correlational tractography in overweight men showed only white matter fibers with a negative relationship with BMI (FDR < 0.001), while men with normal BMI had fibers with both positive and negative relationships with BMI (FDR < 0.01). On the other hand, correlational tractography in normal and overweight women only revealed fibers with a positive relationship with BMI (FDR < 0.001 and FDR < 0.001 respectively), while obese women showed multiple white matter fiber tracts with a negative relationship between connectivity and BMI (FDR < 0.001) (Table 3). Overall, the negative relationship between white matter connectivity and BMI in men was mainly driven by the overweight participants, while the positive relationship between BMI and WM connectome in women was limited to the normal and overweight categories. Due to the low number of participants in the underweight category, we were unable to implement a correlational tractography model separately to men and women in this group.

**Table 3:** Summary of models investigating the relationship of white matter connectivity with BMI in different sex and BMI categories

		Men		Women
	Tracts with a *positive* correlation with BMI	Tracts with a *negative* correlation with BMI		Tracts with a *positive* correlation with BMI	Tracts with a *negative* correlation with BMI
Normal Weight^*^ (*n* = 27)	•Bilateral corticospinal	•Right corticostriatal	Normal Weight^*^ (*n* = 36)	•Bilateral IFOF	–
	•Bilateral corticopontine	•Right IFOF		•Bilateral AF
	•Bilateral SLF	•Right AF		•Bilateral anterior thalamic radiation
	•Left AF			•Bilateral reticulospinal
	•Left IFOF			•Body of corpus callosum
	•Right cingulum			•Left ILF
	•Right reticulospinal			•Right SLF
	•Right dentatorubrothalamic tract			•Right corticospinal
				•Right corticostriatal
				•Right dentatorubrothalamic tracts
r_s_^§^	0.41	0.57	r_s_^§^	0.83	0.42
Overweight^*^ (*n* = 55)	–	•Bilateral IFOF	Overweight^*^ (*n* = 42)	•Bilateral SLF	–
		•Bilateral ILF		•Bilateral anterior thalamic radiation
		•Bilateral SLF		•Bilateral reticulospinal
		•Bilateral corticospinal		•Parahippocampal cingulum
		•Bilateral corticopontine		•Body, tapetum and forceps major of corpus callosum
		•Bilateral corticostriatal	
		•Bilateral anterior thalamic radiation		•Right corticospinal
		•Body and tapetum of corpus callosum		•Right corticopontine
				•Right corticostriatal
		•Left AF
		•Right reticulospinal
		•Right dentatorubrothalamic
r_s_^§^	0.44	0.57	r_s_^§^	0.8	0.85
Obese &Morbidly Obese^*^ (*n* = 22)	•Left corticospinal	•Bilateral SLF	Obese &Morbidly Obese^*^ (*n* = 42)	–	•Bilateral IFOF
	•Left corticopontine	•Right parahippocampal cingulum			•Bilateral ILF
	•Left dentatorubrothalamic	•Left AF			•Bilateral corticostriatal
	•Right IFOF	•Left ILF			•Right corticospinal
	•Right ILF	•Left IFOF			•Right corticopontine
	•Right SLF			
					•Right reticulospinal			
					•Right dentatorubrothalamic			
					•Body and forceps major of corpus callosum
r_s_^§^	0.69	0.79	r_s_^§^	0.76	0.74

^*^All models consisted of a simple or partial Spearman rank-based correlation with local connectivity as dependent variable. Each row demonstrates white matter regions where connectivity was associated with BMI. The false discovery rate was set to 0.05 for all models. BMI categories were defined as normal weight (BMI: 18.5–24.9 kg/m^2^), overweight (BMI: 25–29.9 kg/m^2^), obese & morbidly obese (BMI≥30 kg/m^2^). ^§^Spearman’s correlation coefficient. BMI, body mass index; MMSE, Mini-Mental State Examination; ILF, inferior longitudinal fasciculus; IFOF, inferior frontooccipital fasciculus; SLF, superior longitudinal fasciculus.

### The relationship between BMI and white matter connectivity is not affected by pre-clinical AD status

We further evaluated a subgroup of the study population for whom AD imaging or CSF biomarkers were available within 24 months of dMRI data acquisition (*n* = 210) (Fig. 1). This subset included 56 biomarker positive (30 men and 26 women) and 154 biomarker negative (71 men and 83 women) participants (Table 1 middle and bottom panel). Among biomarker positive participants, 49 participants were amyloid-β positive and 7 were CSF pTau/Aβ_42_ positive. Biomarker positive participants were more likely to be *APOE* ɛ4 carriers and had lower MMSE scores compared to biomarker negative participants. Also, women and biomarker negative participants were more likely to be obese compared to men and biomarker positive participants respectively (Table 1 middle and bottom panel).

We performed correlational tractography separately in biomarker positive and negative men and women (Table 4). Biomarker positive and biomarker negative participants both showed a negative relationship between BMI and connectivity in the bilateral corticopontine tracts, bilateral ILF, bilateral frontoparietal cingulum, and the right dentatorubrothalamic and corticospinal tracts, in men (FDR < 0.01) (Table 4). Correlational tractography in biomarker positive and biomarker negative women revealed a positive relationship between BMI and connectivity in the left corticospinal and the right dentatorubrothalamic tracts (FDR = 0.013 and 0.016, respectively). The pattern of BMI relationship with WM connectome in the biomarker positive and negative subgroups was similar to that of the entire study sample with a negative relationship between BMI and connectivity in men and a positive relationship between WM connectivity and BMI in women (Table 4). There was no statistically significant difference in WM connectivity between biomarker negative and positive participants in neither men nor women. Predictably, an interaction term between CSF biomarker status and BMI did not show any significant relationship with WM connectivity in neither men nor women.

**Table 4:** Summary of models investigating the relationship of white matter connectivity with BMI in biomarker positive and negative participants

Group ^*^	r_s_^§^	Tracts with *negative* relationship of connectivity with BMI in *men* (*n* = 101)			
Biomarker negative (*n* = 71)	0.89	•Bilateral corticopontine tracts =	Bilateral IFOF	Bilateral anterior thalamic radiation	Left corticospinal tract
		•Bilateral inferior longitudinal fasciculus	Right parahippocampal cingulum		
Biomarker positive (*n* = 30)	0.84	•Bilateral frontoparietal cingulum
		•Right dentatorubrothalamic tract
		•Right corticospinal tract
Group ^*^	r_s_^§^	Tracts with *positive* relationship of connectivity with BMI in *men* (*n* = 101)					
Biomarker negative (*n* = 71)	0.56	–	–				
Biomarker positive (*n* = 30)	0.5		Bilateral corticostriatal tracts	Bilateral reticulospinal tracts	Bilateral dentatorubrothalamic tracts	Bilateral SLF	Tapetum of corpus callosum
Group ^*^	r_s_^§^	Tracts with *negative* relationship of connectivity with BMI in *women* (*n* = 109)				
Biomarker negative (*n* = 83)	0.66	–	Bilateral IFOF	Bilateral ILF	Tapetum of corpus	Left anterior thalamic
callosum	radiation
Biomarker positive (*n* = 26)	0.62	–	–			
Group ^*^	r_s_^§^	Tracts with *positive* relationship of connectivity with BMI in *women* (*n* = 109)			
Biomarker negative (*n* = 83)	0.72	•Left corticospinal tract	Left corticobulbar tract	Left corticopontine tract	Right anterior thalamic radiation
		•Right dentatorubrothalamic tracts
Biomarker positive (*n* = 26)	0.7			–

^*^All models consisted of a of a simple or partial Spearman rank-based correlation with local connectivity as dependent variable. Each row demonstrates white matter regions where connectivity was associated with BMI. The false discovery rate was set to 0.05 for all models. Biomarker status was determined based on amyloid beta PET imaging or the CSF amyloid-β peptide 42 measurements as explained in the methods section. ^§^Spearman’s correlation coefficient. BMI, body mass index; MMSE, Mini-Mental State Examination; ILF, inferior longitudinal fasciculus; IFOF, inferior frontooccipital fasciculus; SLF, superior longitudinal fasciculus.

## DISCUSSION

Our analyses revealed distinct relationship patterns between BMI and WM connectivity in men and women. We demonstrated: 1) a statistically significant relationship between higher BMI in *overweight* and *obese* men and *decreased* structural connectivity in the associational fibers of the temporal lobe; namely the IFOF and ILF, 2) a statistically significant relationship between BMI in *normal* to *overweight* women and *increased* WM connectivity in the bilateral anterior thalamic radiations and the corticofugal motor pathways on the right, 3) an inverse relationship between BMI and WM connectome in *obese* and *morbidly obese* women in the bilateral IFOF, bilateral ILF and the corticofugal motor pathways on the right, and finally 5) a similar pattern in the association of WM connectivity in men and women with preclinical AD compared to their biomarker negative counterparts. Our findings collectively suggest that WM connectome is affected by a complex interplay of tissue adiposity and sex. While overweight, obese and morbidly obese men demonstrate detrimental effects of higher BMI in WM connectivity, these effect are not visible in women until they are obese or morbidly obese. Higher tissue adiposity conferred a protective effect on WM connectome in normal to overweight women and potentially due to the neuroprotective effects of estrogen on WM, as discussed later [35].

### Sex as a biological modifier of tissue adiposity, white matter microstructure, and cognition

Mid-life overweight and obesity are well-demonstrated risk factors of late-life dementia [30, 53, 54]. This effect is shown to be independent of modifiable and non-modifiable risk factors for dementia including apolipoprotein E4 carrier status and cardiovascular risk factors, and is more prominent in obese compared to overweight individuals [53]. Age can heavily modify this effect, as evidenced by the inverse U-shape pattern of the relationship between BMI and cognition, with the point of return happening around 65 years old [55, 56].

Despite its significant effects on both BMI [57] and cognition [57], sex as a biological modifier is relatively absent in the above literature and instead often used as a confounder in regression equations. Women are shown to be resistant to the adverse effects of BMI on cognition [58], and a recent report has shown a further risk-reducing effect for BMI on the cognitive ability in older women [59]. Differences in body composition, visceral fat distribution, glucose and lipid homeostasis, and sex steroid hormone levels in premenopausal women are all among suggested mechanisms by which sex can modify the effect of tissue adiposity on cognitive health [60]. Moreover, sex can modify the development of WM in a manner that confers a resistance to mid-adulthood cardiometabolic risk factors to women as discussed in the following sections.

### Premenopausal metabolic profile confers protection to the female white matter

While we did not collect data on hormonal therapy status of the postmenopausal women participants of our cohort, it is important to briefly address potential mechanisms by which hormone status in women can alter the obesity relationship to WM. Menopause is associated with reduced insulin sensitivity and a redistribution of visceral adipose tissue in favor of upper body and abdominal adiposity both of which are associated with reduced white and grey matter integrity [2, 61, 62]. Inversely, when matched for BMI, premenopausal women have an overall higher total body insulin sensitivity compared to men, which is in turn related to improved WM microstructural integrity [63, 64]. Similarly, sex hormone administration to post-menopausal women results in a more favorable visceral fat distribution as well as improved WM volume in this group [65, 66]. As similar effect could not be observed with testosterone administration in older men, inferring that the protective effects of sex hormones on WM integrity and cognition are limited to women [66, 67]. Here we demonstrated a similar resistance in the WM connectome in overweight women that was comparatively reduced in men. Importantly, all but three women in our study cohort were post-menopausal and only twenty-one were in their perimenopausal period when a protective carryover metabolic effect exists up to 6 years after [68, 69]. The positive association between WM connectome and BMI in our female participants therefore cannot be justified merely based on the protective premenopausal metabolic factors. In fact, obesity is associated with increased risk of early menopause and postmenopausal women with obesity have lower levels of estrogen compared to their normal weight counterparts [70, 71]. As a result, the protective effect of premenopausal state on WM health may become undermined by the adverse effect of early menopause and lower post and perimenopausal estrogen levels in obese patients, an effect that is absent in normal to overweight women. On the other hand, the protective premenopausal milieu might have a longer carryover effect on WM integrity in normal or overweight women, conferring a WM protective effect to BMI in these two groups. A similarly long-haul effect is seen with the effect of most mid-life markers of cardiovascular and metabolic health on WM integrity in old age [70, 71]. Finally, the paradoxical re-appearance of several WM tracts with a positive association of connectivity with BMI in obese and morbidly obese men could be justified by increased production of endogenous estrogen by the excess visceral adipose tissue [72].

### Developmental sex differences might alter white matter’s resilience to mid-life metabolic risk factor

Sex difference in WM microstructure can be traced back to early childhood when boys and girls demonstrate tract-specific differences in WM development as early as two years of age [73, 74]. Puberty accentuates these sex-specific trajectories for WM development and is assumed to be the latest time point by which sex affects the WM in a microstructural level [75]. While mentioned effects during childhood and adolescence are mostly ascribed to differences in sex hormones levels, at least part of the sexual-dimorphisms in WM structure are driven by a sex-hormone level independent effects of sex chromosome genes and the pre- and early post-natal hormonal environment [76, 77]. The existence of sex-specific trajectories in WM development can also explain the observed patterns in the association between BMI and WM connectome, whereby tracts that undergo later maturation are more resistant to the age-related changes in integrity [67, 78–80] and the additive effect of obesity on these changes as a result [17]. Later maturation of corticofugal motor tracts and earlier maturation of associational fibers in adolescent girls could therefore underlie the observed positive association of WM connectome in motor tracts with BMI in normal to overweight women and the adverse effect of BMI on WM connectome in long-distance associational fibers of overweight to obese men in our study [74].

### Late-myelinated, long-distance cortico-cortical association fibers mediate the effect of BMI on cognition

As demonstrated in Table 2, the ILF, IFOF, and SLF were frequently identified in the relationship between WM connectivity and BMI. These are among long-distance cortico-cortical associational fibers reaching earlier myelination in women compared to men by 6–7 years [43, 78]. Earlier maturation renders these fibers more vulnerable to age-related microstructural changes as described earlier [78]. Obesity can also affect the microstructure of ILF at a young age, with lower FA and higher MD observed in obese children and those with lower cardiorespiratory and muscular fitness [81, 82]. These effects continue through adulthood, where higher BMI is associated with reduced FA in the bilateral ILF and IFOF independent of the effect of age or cardiovascular risk factors [83]. These observations are in line with our findings of a negative relationship between ILF connectivity and BMI in men, irrespective of age, MMSE score, or AD biomarker positivity. A similar negative association was observed between connectivity in the left ILF and BMI in women with MMSE as a covariate.

Our findings regarding the influence of BMI on connectivity in the IFOF differed significantly by sex. Where men demonstrated a significant negative association between connectivity in the right IFOF and BMI, women showed a significant positive influence that appeared after correction for age. Obese adolescent boys, but not girls, have shown to have increased WM integrity in the right IFOF demonstrated through higher along tracts FA and lower MD [3]. As higher BMI potentiates age-related reduction in WM microstructural integrity, obese boys are expected to have a steeper decline in the right IFOF connectome, hence justifying the negative association with BMI in our older adult cohort. The interaction between age and BMI in predicting lower FA can also explain why the positive relationship between right IFOF connectivity and BMI in adult women appeared only after correction for age (Table 2).

In contrast to the right IFOF, BMI had a significant negative relationship with left IFOF connectivity in both men and women. Decreased WM integrity in the left IFOF is seen in overweight/obese adolescent boys and in obese compared to normal weight young women [84]. Importantly, the IFOF is an important projectional fiber connecting the orbitofrontal cortex and part of the brain’s taste-reward system, which is shown to have lower connectivity in obese young women compared to their normal weight counterparts [6]. Reduced connectivity in the bilateral IFOF is also associated with decreased ability to delay gratification in adolescents [85]. Together these findings suggest that alterations in the integrity of IFOF, especially the left IFOF, might disinhibit the taste-reward circuitry and reduce the ability to delay gratification in favor of food overconsumption and obesity. In this context, our findings regarding the association of IFOF connectivity and BMI are important for two reasons: 1) we illustrate how lower white matter connectivity is related to the behavioral phenomena that contribute to obesity during adolescence, and 2) we show how higher BMI that persists through adulthood results in increasingly lower integrity of the left IFOF in overweight/obese individuals forming a positive feedback loop that further reduces the ability to control food overconsumption. The latter is supported by the significant effect of the interaction between BMI and age in reducing the FA in the bilateral IFOF [3]. Finally, both ILF and IFOF are implicated in a variety of cognitive functions, suggesting a potential link between higher BMI and cognitive decline in our older adult cohort [86–88].

The corticofugal motor pathways; namely the corticopontine tracts were also among fibers where connectivity negatively related to BMI in men but positively in women. A review of literature reveals a dimorphism among adolescent boys and girls in the association of lifestyle-related factors and microstructure of the corticospinal tracts. In adolescent boys, aerobic fitness is associated with decreased FA in the left corticospinal tract [89] and obesity is linked with increased FA in the bilateral corticospinal tracts [3]. Obese adolescent girls, however, have a lower FA compared to their normal weight counterparts [3]. During adulthood, both obese men and obese women demonstrate an increase in MD and apparent diffusion coefficient, and a decrease in FA in the corticospinal tracts [90–92]. The observed dichotomy in the direction of association during adolescence might inform developmental sex differences that disappear with the continued effect of obesity in WM connectome in adult men and women.

The tapetum of the corpus callosum and the frontoparietal and parahippocampal divisions of the cingulum were another set of fibers with inverse relations to connectivity and BMI in men and positive association of connectivity with BMI in women. The tapetum of the corpus callosum connects the right and left temporal lobes and the parahippocampal and frontoparietal parts of the cingulum connect the anterior and posterior cingulate cortices to the medial temporal lobe [93]. Components of the medial temporal lobe belong to the taste-reward circuitry that is known for its function in hedonic control of food intake [5]. Moreover, the anterior cingulate cortex functions in the salience and emotional arousal neural networks [6]. Our results therefore extend on the observations showing that altered communication between the salience and reward circuitry might give rise to eating habits that predispose to obesity [94].

Finally, our analyses indicated a significant positive relationship between connectivity in bilateral SLF and BMI in women. This observation is in line with earlier reports demonstrating increasing axial diffusivity in the SLF with increased BMI in adult participants [95], but opposes later reports showing a decrease in FA on left SLF with increasing BMI in adolescents [81]. SLF has been connected to language function, mentalizing ability, and self-face/body recognition [87]. In line with the later function, lower FA in bilateral SLF is seen both in young adult women suffering from anorexia nervosa and binge eating disorder [96, 97]. In view of these findings, we speculate that the observed positive relationship between SLF connectivity and BMI in adult women implicates a positive hedonic experience with eating during participant’s adolescence, potentially resulting in food overconsumption. Similar to the right IFOF, an age-specific difference is seen in the direction of the association between microstructural integrity of the SLF and BMI. This could explain why the relationship between SLF connectivity and BMI only appeared after correction for age.

### Strengths, limitations, and future directions

The diffusion MRI connectometry analyses used in this research come with inherent strengths compared to conventional diffusion MRI analytic models, namely the diffusion tensor imaging (DTI). The QSDR is a generalization of the Generalized Q-sampling Imaging (GQI) model. GQI is a model-free diffusion reconstruction method that adopts the density of water diffusion in different directions, rather than *diffusivity* that is used by DTI, as the main function of interest. The QSDR outputs the SDF, which is an orientation distribution function of diffusing spins. SDF is then sampled at each peak orientation to yield a QA value for each fiber orientation [45]. Unlike conventional diffusion metrics such as FA which are voxel-defined, QA is defined for each fiber orientation, giving the QSDR a unique ability to filter out false trajectories in areas with crossing fiber [44]. As both SDF and QA have arbitrary units it is important to normalize the QA before using this metric to assess any inter-subject variability. This was achieved in our analytic approach by scaling the maximum QA value of a subject to 1 [44]. Finally, the non-restricted and restricted diffusion are often mixed in the DTI model due to Brownian motion, whereas the GQI and QSDR methods quantify diffusion *density* that simplifies the separation of restricted and non-restricted diffusion and increases the performance in low signal-to-noise ratio conditions compared to DTI.

The heterogeneity of the study population in terms of their preclinical AD status, was a major limitation of this study. This was addressed by determining biomarker positivity through CSF and amyloid PET and subgroup analyses based on biomarker status. Nonetheless, the 24-month allowed interval between CSF or PET amyloid measurement might result in misclassification of participant in the biomarker positive or negative groups. Similarly, cognitive scores including MMSE and CDR were measured during a clinical visit within 12 months of the diffusion MR study and not on the same day as the diffusion MR studies. To address this, the absolute time difference between the dates of clinical assessment and dMRI acquisition was added as a covariate to all the models that included the MMSE score. Finally, we used BMI as a simple and accessible measurement of total body adiposity. However, BMI is not sufficient for optimal assessment of metabolic risk factors that are associated with obesity and their potential effect on brain health [98]. Therefore, when evaluating the relationship between BMI and brain aging it is prudent to consider other lifestyle factors such as diet, smoking, and physical activity which have all been shown to significantly impact aging and mortality in large population-based cohorts [99]. Further studies are warranted to address the association of WM connectome with more specific metrics of tissue adiposity such as visceral fat measurement through dual-energy X-ray absorptiometry, abdominal CT or MRI while taking other such risk factors into account.

In summary, our results support the hypothesis that BMI affects WM structural connectivity differently in men and women, and that this relationship is not altered in the setting of preclinical AD. When viewed in the context of existing literature, our findings imply that sex differences in the reward and emotional arousal networks [100] give rise to altered connectivity in the WM tracts associated with these networks as well as different neuronal underpinnings for obesity between men and women. Adding the interaction of BMI and age that alters the microstructural integrity of certain WM tracts, a positive feedback loop forms where persistent high BMI alters the connectome of temporal lobe associational WM fibers more prominently in men than women. Moreover, increased vulnerability in men of the WM connectome to obesity, along with implications of these WM fibers in cognition, inform a male-specific association of obesity and cognition. This provides a target for future studies to investigate the effect of lifestyle modifications to combat adolescent and adulthood obesity in reducing life-long risk of dementia.
